# The impact of new VAT enforcement on financial performance: Evidence from Saudi Arabia non-financial listed companies using the event study and ARMA model

**DOI:** 10.1016/j.heliyon.2024.e39137

**Published:** 2024-10-10

**Authors:** Mohammed Ibrahim Al-Otaibi, Normaziah Mohd Nor, Yusniyati Yusri, Nedaa Guzaiz

**Affiliations:** a University of Putra Malaysia, School of Business and Economics, 43400, Serdang, Selangor, Malaysia; b Umm Al-Qura University, College of Business and Economics, Makkah, Kingdom of Saudi Arabia

**Keywords:** Value-added tax (VAT), Event study, Non-financial sectors, Autoregressive moving average (ARMA) model, Economic impact, Financial stability

## Abstract

This paper examines the influence of the new 15 % Value Added Tax (VAT) enforcement on non-financial listed companies in Saudi Arabia. By comparing financial data from 2019, before the VAT implementation and the COVID-19 pandemic, with data from 2020, during both the VAT increase and the pandemic, the research aims to uncover the consequences of this tax policy change. Utilizing charts, tables, and an event study analysis approach with the Autoregressive Moving Average (ARMA) model, we investigated key financial indicators such as Shareholders' Equity (SE), Total Income (TI), Total Revenues (TR), Net Income (NI), Total Expenses (TE), Other Changes in Operating Activity (COA), and Cash at the End of the Period (CEP). The findings reveal significant financial impacts, highlighting companies' challenges due to the increased tax burden. Shareholders' Equity dropped from an average of 11.55 million to 4.57 million, and Total Income and Total Revenues decreased from approximately 1.73 million to 316,234 and from 1.95 million to 331,605, respectively. Net Income sharply declined from 406,109 to 74,624, and Total Expenses decreased from about 1.18 million to 223,495. Other Changes in Operating Activity shifted from a negative mean of −168,936 to a positive 523,520, and Cash at the End of the Period fell from 1.99 million to 666,663. These results suggest that the VAT increase has significantly strained companies' financial performance, emphasizing the need to consider such tax reforms carefully, particularly in developing countries. The study concludes that while the VAT increase aims to enhance government revenues, it imposes substantial financial strain on companies, potentially leading to long-term economic repercussions. Policymakers should consider these implications and implement practical measures to optimize the VAT system and support business resilience in the face of such reforms.

## 1 Introduction

Widespread taxation reforms across industrialized and emerging economies characterize contemporary global economic landscapes. These reforms are driven by various factors, including macroeconomic conditions, shifting governmental priorities, and responses to emerging socioeconomic challenges [1]. The impact of tax policies on various sectors is a subject of ongoing research. For instance, Adedoyin et al. [2] examine the correlation between tax policies and international tourism in the Maldives, while Helga [3] investigates the implications of VAT increases on tourism demand in Europe. Taxation remains a fundamental instrument for government revenue generation and is pivotal in fostering economic growth and development. An effective tax system incorporates a balance of direct and indirect taxes, contributing to a nation's overall fiscal stability and sustainability [4]. Nevertheless, in numerous emerging economies, direct taxation often diminishes revenue for investments' public sector [5]. Consequently, these markets frequently exhibit a greater faith in indirect taxation mechanisms, such as value-added taxes (VAT) or goods and services taxes (GST), to finance public sector expenditures [6].

The Saudi Arabian government implemented a 5 % Value Added Tax (VAT) to diversify its revenue streams on January 1, 2018. Subsequently, in response to the economic repercussions of the COVID-19 pandemic and declining oil prices, the government announced, on May 11, 2020, a tripling of the VAT rate to 15 %, effective July 1, 2020 [7]. To mitigate the financial burden on citizens resulting from VAT implementation and rising fuel costs, a monthly allowance of 1000 riyals ($267; £217) was introduced for state employees in 2018. The VAT-generated revenue is intended to support the sustained obligation of high-quality public services and aligns with Saudi Arabia's Vision 2030. The introduction of VAT is anticipated to boost reporting simplicity and corporate culpability, potentially leading to more robust and formalized internal documentation practices within Saudi Arabia. While the VAT's implementation is expected to yield various positive outcomes, it will likely have differential impacts across economic sectors. This study investigates the effects of VAT implementation on non-financial companies listed in the Saudi stock market. It seeks to contribute to the existing literature by examining the implications of VAT introduction specifically within the context of Saudi Arabia, an emerging market historically characterized by minimal reliance on indirect taxation. The research has three primary objectives: first, To quantify the impact of the VAT implementation on critical financial metrics of non-financial companies in Saudi Arabia; second, to explore how VAT design influences corporate profitability and economic growth dynamics in the short and long term; and third, to provide empirical evidence on the implications of VAT rate restructuring for policy considerations in Saudi Arabia and other Gulf Cooperation Council (GCC) nations.

The principal scholarly contributions of this research can be presented as follows.

a) This study contributes uniquely to VAT and taxation literature by examining the impact of increasing VAT to 15 % on non-financial companies in Saudi Arabia, filling a notable gap in existing research.
b) Utilizes detailed, disaggregated data and employs advanced analytical methods, including event study and autoregressive integrated moving average (ARIMA) method, to establish robust correlations over two distinct periods.
c) Demonstrates that while a VAT increase initially decreases corporate profitability in Saudi Arabia, it can lead to long-term benefits such as increased government revenue and potential economic stimulus through investment.
d) Provides empirical and theoretical groundwork for refining VAT policies to encourage sustainable economic growth and reduce regional economic disparities.

The remainder of the paper is organized as follows: Section 2 reviews the existing literature; Section 3 outlines the tax system in the Kingdom of Saudi Arabia (KSA); Sections 4, 5 describe the dataset and methodology; Section 6 presents the results and discussion; and Section 7 offers the conclusion.

## 2 Survey of literature

Introducing fiscal measures encompassing direct and indirect taxation mechanisms significantly influences economic growth trajectories and investment patterns. The Efficient Market Hypothesis (EMH) posits that financial markets exhibit high efficiency in assimilating and reflecting all available information, thereby rendering consistent attainment of above-average returns through active trading strategies largely infeasible. The EMH postulates that asset valuations perpetually incorporate all known information, thus significantly impeding investors' capacity to exploit perceived market inefficiencies or asset mispricing [8]. Consequently, financial markets are theorized to respond with celerity to introducing novel information. This implies that any modifications to tax regulatory frameworks are expected to elicit prompt market reactions. These market responses, which garner considerable attention from academic researchers, industry practitioners, and policy formulators, serve as crucial indicators for assessing the efficacy and impact of tax policies. The rapidity and magnitude of these market reactions provide valuable insights into the perceived implications of tax policy changes, offering a quantifiable metric for evaluating the anticipated economic consequences of fiscal interventions. This dynamic interplay between tax policy implementation and market response mechanisms underscores the importance of rigorous analysis in understanding the broader economic ramifications of taxation strategies.

Value Added Tax (VAT) is a consumption-based tax imposed on the added value of goods and services at every stage of production or distribution. This tax is usually collected at each step of the supply chain, where businesses charge VAT on the goods and services they provide and then remit the collected tax to the government. Since the final consumer ultimately pays VAT as part of the purchase price, it is classified as an indirect tax [9]. The VAT system aims to tax consumption rather than income or profits, making it a more equitable and efficient way to generate government revenue. It is widely used worldwide by both developed and developing countries [10]. Registered businesses assume the role of tax collectors on behalf of the government by collecting and accounting for the tax. In this capacity, they are responsible for collecting the applicable taxes from their customers or clients and remitting them to the government as required [11]. This system delegates the task of tax collection to businesses, which then transfer the collected taxes to the government, effectively serving as intermediaries in the tax collection process [12]. Value-added tax (VAT) proponents often advocate for its flat rate structure for several reasons [9]. Firstly, a flat rate simplifies the tax system, making it easier to understand and administer for taxpayers and tax authorities. With a single rate applying to most or all goods and services, there's less complexity in calculating and collecting taxes. Secondly, a flat VAT rate can promote fairness and equity in taxation. Since everyone pays the same percentage of tax on their purchases regardless of their income level, it can be seen as a more equitable way to distribute the tax burden across society. Moreover, a flat rate minimizes distortions in consumption behaviour. When different goods and services are taxed at varying rates, it can lead to market inefficiencies and distortions. Consumers may alter their purchasing decisions based on tax considerations rather than purely on their preferences or needs. A uniform VAT rate avoids such distortions, allowing market forces to operate more efficiently. Additionally, a flat VAT rate can enhance government revenue stability. Governments can more accurately forecast and plan their revenue streams by having a predictable and consistent tax rate across all sectors, aiding in budgetary management and long-term fiscal planning. VAT is a significant source of government revenue in many countries due to its broad base and relatively efficient collection mechanism. One of the key features of VAT is its inflation-indexed nature, which means that the tax rate can be adjusted to account for changes in the general price level, helping to maintain its real value over time. This indexing ensures that VAT remains an effective government revenue generator, even during periods of inflation or deflation [13]. Additionally, VAT is often considered self-policing against tax avoidance or evasion to some extent due to its built-in mechanisms for accountability and transparency. Unlike income tax, where individuals or businesses may underreport income or engage in other forms of evasion, VAT operates at each production and distribution chain stage, allowing for greater visibility and control over transactions [14]. VAT helps combat tax avoidance through its invoice-based system, where businesses must document and report their transactions, including the VAT they collect and pay.

This paper trail makes it more difficult for businesses to conceal their sales or purchases, reducing opportunities for tax evasion. Moreover, input tax credits further strengthen VAT's self-policing mechanism. Businesses can typically deduct the VAT they pay on purchases (input tax) from the VAT they collect on sales (output tax), effectively paying tax only on the value they add to goods or services. This feature encourages businesses to ensure that their suppliers also comply with VAT regulations, as they can only claim input tax credits if their suppliers have properly charged and remitted VAT [13,14]. The existing literature indicates that VAT is designed to be a more efficient and neutral form of taxation than traditional sales taxes, as it minimizes the distortions and inefficiencies associated with tax cascading. By allowing businesses to claim input tax credits, taxing only the value added at each stage, and incorporating border adjustments, VAT helps to ensure a more equitable and transparent tax system that promotes economic efficiency and competitiveness [15]. Conversely, opponents of VAT often argue that these drawbacks outweigh the purported benefits of the tax, such as its revenue-raising potential and compatibility with international trade. They may advocate for alternative tax policies or reforms to reduce the compliance burden and mitigate the inflationary impact of indirect taxation. Additionally, critics may highlight the regressive nature of VAT, as lower-income individuals tend to spend a higher proportion of their income on consumption, making them disproportionately affected by price increases resulting from VAT implementation [16]. Pinheiro et al. [17] report on taxpayer resistance to introducing VAT underscores an important aspect of tax policy implementation. Tax reforms, including introducing new taxes such as VAT, can often face opposition and resistance from various stakeholders, including taxpayers, businesses, and interest groups. However, there is limited research on the relevance of VAT for emerging markets, and our paper seeks to address this gap in the literature.

Critics have pointed out that Value Added Tax (VAT) can harm corporate performance, leading to decreased sales volume and subsequent wealth reduction [18]. From an economic standpoint, Thomas [19] examines the regressive quality of VAT, highlighting its failure to address inequality. Moreover, Sebele et al. [20] argue that the substantial informal sector in emerging economies poses challenges to the efficiency of VAT in these settings. On the other hand, other studies propose that VAT implicitly taxes inputs and imports applied by the informal sector [21]. According to Chen et al. [22], while the introduction of VAT can potentially lead to increased government spending, investment, and economic growth, its impact is contingent upon the design and implementation of the tax and the overall economic environment in which it is applied. Whether VAT improves economic well-being remains a nuanced and ongoing question that requires careful consideration of its effects across different segments of society.

Much of the research on taxation tends to focus either on its overarching economic effects or its social implications. This research often employs methodologies rooted in economic theory, such as equilibrium and welfare analyses, or utilizes descriptive qualitative approaches, as exemplified in Ref. [23]. An exceptional instance is provided by Husain et al. [24], who employ the event study approach to investigate the correlation between Sukuk issuance and various macroeconomic indicators. In this regard, the body of literature examining the responses of the non-financial market to the implementation of indirect taxes is notably limited, particularly in developing nations like Saudi Arabia. Our study adds to this area of research.

## 3 Tax system in KSA

The tax system in Saudi Arabia has undergone substantial transformations as part of the country's Vision 2030 initiative, which aims to diversify the economy and reduce its dependency on oil revenue. Traditionally, Saudi Arabia did not impose an income tax on individuals, focusing instead on corporate taxes and foreign investments. This historical absence of personal income tax has been a significant aspect of the country's fiscal policy, making it attractive for expatriates and multinational corporations operating within its borders [25]. One of the most notable changes in the Saudi tax landscape was adding value-added tax (VAT) in 2018. Initially set at 5 %, the VAT rate tripled to 15 % in 2020 due to economic challenges exacerbated by the COVID-19 pandemic and fluctuating oil prices [25]. VAT is applied to most goods and services, excluding essentials such as food, healthcare, and education, and it has become a critical source of non-oil revenue for the government [25]. The introduction of VAT marked a significant shift, requiring businesses to overhaul their accounting systems and ensure compliance with the new regulations. Corporate taxation in Saudi Arabia is primarily directed at foreign entities and non-Saudi businesses. The standard corporate income tax rate is 20 % on profits generated within the kingdom [26]. This taxation framework ensures that foreign companies contribute fairly to the Saudi economy. In addition to corporate income tax, the government imposes Zakat, a religious wealth tax, on Saudi and GCC (Gulf Cooperation Council) nationals and businesses [26]. Zakat is calculated at 2.5 % of the company's net worth and serves religious and fiscal purposes, integrating traditional Islamic principles with modern economic policies [26].

Saudi Arabia has invested heavily in modernizing its tax administration to enhance compliance and efficiency in tax collection. The General Authority of Zakat and Tax (GAZT), which has now merged into the Zakat, Tax, and Customs Authority (ZATCA), oversees tax collection and enforcement. ZATCA has introduced various digital platforms to streamline tax registration, filing, and payment processes [25]. This digital transformation aims to make it easier for businesses and individuals to fulfil their tax obligations while increasing transparency and accountability within the tax system [25]. Implementing VAT and the modernization of tax administration have had significant implications for businesses operating in Saudi Arabia. Companies have had to adapt to new compliance requirements, invest in updated accounting software, and train staff to handle VAT-related procedures [27]. This shift has been particularly challenging for small and medium-sized enterprises (SMEs), which may have limited resources to manage the additional administrative burden [27]. However, these changes are necessary to create a more robust and sustainable fiscal environment.

The introduction of excise taxes in 2017 also represents a significant development in Saudi Arabia's tax policy. Excise taxes are levied on goods considered harmful to health or the environment, such as tobacco, sugary drinks, and energy drinks. These taxes aim to reduce the consumption of these products and generate additional revenue for public health initiatives. The rates for excise taxes vary, with some products facing up to 100 % tax, reflecting the government's commitment to addressing public health concerns and promoting healthier lifestyles [28]. Foreign investments in Saudi Arabia are subject to specific tax regulations designed to attract and regulate international business activities. In addition to the corporate income tax, foreign investors may be subject to withholding taxes on payments such as dividends, interest, and royalties. These taxes are typically set at a rate of 5 %–20 %, depending on the nature of the payment and the applicable double taxation treaties [25]. Saudi Arabia has entered into numerous bilateral tax treaties to avoid double taxation and encourage foreign investment, enhancing its attractiveness as a business destination [25]. Saudi Arabia's tax system also includes incentives to promote economic growth and diversification. The government offers various tax exemptions and incentives for businesses in specific sectors such as manufacturing, technology, and renewable energy. These incentives are designed to attract investment in industries that align with the goals of Vision 2030, fostering innovation and creating job opportunities for Saudi nationals [28]. The government continuously reviews and updates these incentives to ensure they remain competitive and effectively achieve their objectives. The personal income tax remains absent in Saudi Arabia, maintaining its appeal to expatriates and high-net-worth individuals. However, the government has introduced other levies, such as the expat levy, which charges employers a fee for each foreign worker. This policy encourages hiring Saudi nationals and reduces the kingdom's reliance on expatriate labor. The expat levy has significantly impacted the labor market, prompting some companies to reconsider their workforce strategies and investment plans.

## 4 Dataset

We utilized periodic data from various Saudi-registered companies before implementing the new 15 % VAT. Our study focused on the second and third quarters of 2019 and the third and fourth quarters of 2020. We selected this sample because public information on VAT for non-financial firms is available [29]. Tadawul mandates that publicly traded companies disclose their financial statements quarterly and annually on their website. The Tadawul All Share Index (TASI) comprises 192 companies across 11 key sectors: energy, consumer discretionary, real estate, consumer staples, materials, healthcare, information technology, industrials, communications services, utilities, and finance. Financial institutions were excluded from our analysis due to their distinct transactional nature, and previous studies have already examined the impact of VAT on Saudi banks.

## 5 Methodology

The current research utilizes the Event Study Method (ESM) with the Autoregressive Integrated Moving Average (ARIMA) model as a robust framework for analyzing the impact of discrete events on financial time series data. ESM can pinpoint abnormal returns surrounding specific events, providing insights into immediate market reactions [30]. Meanwhile, ARIMA excels in modeling the underlying time series dynamics, capturing trends, seasonality, and autocorrelation within the data [31]. By integrating these methodologies, analysts can quantify the short-term effects of events and forecast their longer-term implications on asset prices, enhancing the depth and accuracy of financial analysis and decision-making.

### 5.1 Event study method

Event studies analyze the impact of specific events on the value of a firm's stock. Originating in the 1960s, they assess how earnings announcements, mergers, and regulatory changes affect stock prices [30]. By examining abnormal returns and deviations from expected performance, researchers can infer the market's reaction to the event. This method leverages the efficient market hypothesis, assuming that stock prices reflect all available information [30]. Event studies are valuable for understanding market efficiency, investor behavior, and the economic impact of corporate actions. The common approach of an event study begins with defining the event of interest and the event window, which includes the days around the event date. Researchers then select a sample of firms affected by the event and collect data on their stock prices before and after the event. The next step involves estimating each firm's average (expected) returns during the event window, often using a statistical model like the market model, which relates the stock's returns to the overall market returns. The abnormal returns, or the difference between the actual and expected returns, are then calculated and analyzed to determine the event's impact on stock prices [32].

The analysis focused on the second and third quarters of 2019, before implementing a new 15 % VAT and before the onset of COVID-19. Additionally, the study examined the third and fourth quarters of 2020, following the introduction of the new 15 % VAT and during the COVID-19 pandemic. The primary objective was to investigate the presence of abnormal returns during these specified event periods. Findings revealed diverse behaviors among participants in the stock markets of non-financial companies listed in Saudi Arabia. Moreover, VAT increases impact businesses before and after implementation, albeit to differing extents. The analysis begins with applying the market model, as demonstrated in equation (1).

(1) $$R_{i}^{t}=\alpha_{i}+\beta_{i}R_{m}^{t}+u_{i}^{t}$$

in which, $R_{i}^{t}$, and $R_{m}^{t}$ are daily returns of stock i and daily market index return on the stock market m at time t, respectively. $u_{i}^{t}$ represents the residual of stock i at time t, which is independent and identically distributed.

After that, the parameters of the market model are estimated during the estimation window 2019. The abnormal return of stock i at time t is defined in equation (2):

(2) $$AR_{i}^{t}=R_{i}^{t}-{\hat{\alpha }}_{i}-{\hat{\beta }}_{i}R_{i}^{t}$$

in which, ${\hat{\alpha }}_{i}$ and ${\hat{\beta }}_{i}$ are the estimated parameters of stock i from equation (1).

$AR_{i}^{t}$ represents the event's effect when information about the three aforementioned events is disclosed to the markets. Significant deviations of $AR_{i}^{t}$ from zero indicates that market value diverges from its fair value. ${CAR}_{i,\left(t_{1},t_{2}\right)}$ represents an accumulated abnormal return of stock i during the period $t_{1}$ and $t_{2}$. The study examines whether market value diverges from fair value by testing the significance of ${CAR}_{i,\left(t_{1},t_{2}\right)}$. A negative or positive ${CAR}_{i,\left(t_{1},t_{2}\right)}$ indicates that stock prices depart from their fair value during the analyzed period when the market reacts to new information. Mathematically, ${CAR}_{i,\left(t_{1},t_{2}\right)}$ is defined in equation (3):

(3) $${CAR}_{i,\left(t_{1},t_{2}\right)}=\sum_{t=t_{1}}^{t_{2}}AR_{i}^{t}$$

An event window from 7 days before to 7 days after each event date (−7, +7) is employed. This range is chosen to align with the efficiency of stock markets. It is consistent with previous transportation and logistics research studies, which typically limit the event window to no more than 10 days [33]. Furthermore, selecting a shorter event window helps avoid overlapping event periods [34]. Moreover, a longer event window may diminish statistical power [35].

### 5.2 Overview of ARIMA model

The Autoregressive Integrated Moving Average (ARIMA) models have their roots in time series analysis and were popularized through the seminal work of Box and Jenkins in the early 1970s [36]. The Box-Jenkins methodology provided a systematic approach for identifying, estimating, and checking models for time series forecasting. This methodology, which emphasizes making a time series stationary through differencing, laid the foundation for the ARIMA model's widespread application [31]. By combining the autoregressive and moving average components with differencing, ARIMA models offer a robust framework for capturing the underlying patterns in temporal data, making them invaluable for economic, financial, and scientific forecasting [31].

The ARMA models of a time series $y_{t}$ consist of two main components:1) the AutoRegressive (AR) part, which expresses the current value of the series as a linear combination of its previous values (lags) and a stochastic error term; 2) the Moving Average (MA) part, which models the current value of the series as a linear combination of past error terms. Mathematically, it is represented in equation (4):

(4) $$y_{i}=\sum_{i=1}^{p}\varphi_{i}y_{t-i}+\sum_{j=1}^{q}\theta_{j}e_{t-j}$$

in which, $y_{t-i}$, and $e_{t-j}$ represent the lagged past values and errors, respectively. $\varphi_{i}$ is the coefficient for the autoregressive component, while $\theta_{j}$ is the coefficient for the moving average term. p and q are orders and determine the number of coefficient parameters, respectively.

ARMA(p,q) models are suitable for at least weakly stationary series, defined by having a finite and constant mean and variance, and where the covariance between observations from two periods depends solely on the lag. Consequently, proper data preprocessing is crucial when applying this approach to non-stationary series.

The most common method of removing non-stationarity in terms of the mean (e.g., trend) can be integrated with the model. With B as the backward operator to indicate differencing, $B\left(y_{t}\right)=y_{t}-y_{t-1}$, the ARMA(p,q) model with an integration (differencing) d to delete non-stationarity is expressed equations (5), (6), (7):

(5) $$\varphi_{p}\left(B\right){\left(1-B\right)}^{d}y_{t}=\theta_{q}\left(B\right)e_{t}$$

(6) $$\varphi_{p}\left(B\right)=1-\varphi_{1}B-\ldots -\varphi_{p}B^{p}$$

(7) $$\theta_{q}\left(B\right)=1-\theta_{1}B-\ldots -\theta_{q}B^{q}$$

in which, $\varphi_{p}\left(B\right)$ is the moving average operator, represented as a polynomial in the backshift operator; $\theta_{q}\left(B\right)$ is an autoregressive operator, represented as a polynomial in the backshift operator.

The SARIMA(p,d,q)(P,D,Q)s models include seasonality in time series, where s is the number of seasons in the seasonal cycle as shown in Equations (8), (9), (10):

(8) $$\varphi_{p}\left(B^{s}\right)\varphi_{p}\left(B\right){\left(1-B\right)}^{d}{\left(1-B^{s}\right)}^{D}y_{t}=\Theta_{Q}\left(B^{s}\right)\theta_{q}\left(B\right)e_{t}$$

(9) $$\varphi_{p}\left(B^{s}\right)=1-\varphi_{1}B^{s}-\ldots -\varphi_{p}B^{ps}$$

(10) $$\Theta_{Q}\left(B^{s}\right)=1-\Theta_{1}B^{s}-\ldots -\Theta_{Q}B^{Qs}$$

in which, $\varphi_{p}\left(B^{s}\right)$, and $\Theta_{Q}\left(B^{s}\right)$ is the seasonal autoregressive operator and the seasonal moving average operator, respectively.

The ARIMA/SARIMA model describes the $y_{t}$ using the preceding values of the $y_{t}$ and the forecast error $e_{t}$. An extension of the classic ARIMA models includes a set of exogenous series $x_{i,t}$ as input variables and is referred to as an ARIMX in equation (11):

(11) $$y_{i}=\mu +\sum_{i}\frac{\omega_{i}\left(B\right)}{\delta_{i}\left(B\right)}B^{k_{i}}x_{i,t}+\frac{\theta_{q}\left(B\right)}{\varphi_{q}\left(B\right)}e_{t}$$

in which, μ is a constant; $\omega_{i}\left(B\right)$ represents a numerator polynomial of the transfer function for the $i^{th}$ input series; $\delta_{i}\left(B\right)$ is a denominator polynomial of the transfer function for the $i^{th}$ input series; $k_{i}$ is the pure delay for the effect of $x_{i,t}$ at time t.

An appropriate ARIMA model hinges on accurately identifying autocorrelation and partial autocorrelation patterns. A typical method for determining the suitable ARIMA structure involves sequentially comparing models with varying parameters to identify the one that best meets the fit criteria [37].

### 5.3 Integration of ESM with ARIMA model

Integrating event studies with ARIMA models involves a systematic approach to assess the impact of specific events on time series data, such as stock prices or economic indicators. The process begins by identifying the event of interest and collecting relevant time series data surrounding the event date. The event window is then defined, typically divided into pre-event, event, and post-event periods. This segmentation allows for a transparent data comparison before, during, and after the event. An ARIMA model is fitted to the pre-event data to establish a baseline expectation. ARIMA parameters (p, d, q) are selected based on stationarity requirements, and methods such as ACF/PACF plots and information criteria (AIC/BIC) are used for parameter optimization. The model is then applied to predict expected values during the event and post-event periods. The core of the event study involves calculating abnormal returns by comparing actual observed values with those predicted by the ARIMA model. These abnormal returns, which represent the deviation from expected performance due to the event, are aggregated to form cumulative abnormal returns (CAR). Statistical tests, such as t-tests, are conducted to determine the significance of these abnormal returns, thereby assessing the event's impact. Parameter adjustments, such as refining the length of the event windows and selecting appropriate ARIMA parameters and statistical tests, are crucial for enhancing the accuracy and reliability of the results. The final step involves interpreting the results in the event context, where significant CARs indicate a notable impact of the event on the time series. This integration of ARIMA and event studies provides a robust framework for understanding the dynamic effects of events on time series data.

Here's an outline of how to approach this integration:

Step 1: Data Preparation.

Begin by collecting data on historical time series relevant to equity prices or returns for the selected securities over the event and estimation windows. This may involve obtaining historical market prices or other relevant financial data. Identify the date of the event we want to analyze, referred to as $t_{0}$. This step ensures a comprehensive dataset and a clear reference point for the event's occurrence.

Step 2: Define Windows.

Define two critical windows: the estimation window and the event window. The estimation window, such as $\left[t_{0}-100,t_{0}-11\right]$ (e.g., from 100 days before the event to 11 days before the event), precedes the event and is used to estimate the parameters of the ARIMA model. The event window, such as $\left[t_{0}-7,t_{0}+7\right]$ (e.g., from 7 days before the event to 7 days after the event), surrounds the event date and captures the period during which the event's impact is expected to manifest. These windows help isolate the event's effect from the underlying time series patterns. The 14-day event window utilized in this study is effective for capturing short-term market reactions and immediate abnormal returns. However, we acknowledge that this timeframe may not fully capture the companies' long-term financial health or operational efficiency under analysis. Additional analyses with extended event windows may be necessary to assess the broader, long-term impacts of the VAT increase. Nevertheless, the focus of this study remains on short-term market responses, as these provide critical insights into immediate financial adjustments following the VAT implementation.

Step 3: Model Estimation.

Fit an ARIMA model to the data within the estimation window. The ARIMA model helps capture trends, seasonality, and autocorrelation in the time series data. Once the model is fitted, use it to forecast the expected values during the event window. This step provides a baseline against which we can compare the actual data during the event window.

The potential endogeneity of the expected instruments for examining independent variables (IVs) raised concerns, leading to the incorporation of logarithmic variations in VAT. To tackle this challenge, the standard event study model was adaptable through diverse methodologies. The ARMA(p,q) model is typically used to represent weakly stationary stochastic time series in multinomials. An “SARIMA(p,d,q)(P,D,Q)s model” was developed, ensuring the ARIMA model variance aligns with the minimum AIC and BIC criteria to determine specific values of p and q. The ARIMA approach is appropriate for single-dimensional models exhibiting time-dependent fluctuations. Diagnostic procedures involved analyzing data and logarithms for first-difference to achieve stability in series variations. To determine suitable values for autoregression and the moving average (MA) component in the model, the Augmented Dickey-Fuller (ADF) test was utilized. The $ARIMA\;\left(1,0,0\right){\left(0,1,1\right)}_{11}$ model was found effective for time series analysis and time delay forecasting.

Step 4: Calculate Abnormal Returns.

During the event window, abnormal returns are calculated by comparing the actual observed values to the forecasted values from the ARIMA model. Abnormal return for each day t is computed as $AR\left(t\right)=Y\left(t\right)-\hat{Y}\left(t\right)$, where Y(t) is the actual value and $\hat{Y}\left(t\right)$ is the forecasted value. These abnormal returns represent the deviations attributable to the event, providing a measure of its immediate impact. The proposed method also necessitates data from control and treatment groups across two periods: once before and after introducing the “15 % VAT.” Hence, the forecasted value is given as equation (12):

(12) $${\hat{Y}}_{i}\left(tratement=ARIMA\;ESM\right)=\beta_{0}+\beta_{1}TAb+\beta_{2}TAa+\beta_{3}TAb*TAa+\beta_{4}SEb+\beta_{5}SEa+\beta_{6}SEb*SEa+\beta_{7}TLSEb+\beta_{8}TLSEa+\beta_{9}TLSEb*TLSEa+\beta_{10}TIb+\beta_{11}TIa+\beta_{12}TIb*TIa+\beta_{13}TRb+\beta_{14}TRa+\beta_{15}TRb*TRa+\beta_{16}TEb+\beta_{17}TEa+\beta_{18}TEb*TEa+\beta_{19}NIb+\beta_{20}NIa+\beta_{21}NIb*NIa+\beta_{22}COAb+\beta_{23}COAa+\beta_{24}COAb*COAa+\beta_{25}CIAb+\beta_{26}CIAa+\beta_{27}CIAb*CIAa+\beta_{28}CFAb+\beta_{29}CFAa+\beta_{30}CFAb*CFAa+\beta_{31}CEPb+\beta_{32}CEPa+\beta_{33}CEPb*CEPa+\varepsilon_{i}$$

in which, $\varepsilon_{i}$ is an error, and other variable definitions are indicated in Table 1.

**Table 1:** Definition of variables in equation (12).

Variable definition	Balance sheet		Statement of income		Cash flow	
	Before	After	Before	After	Before	After
Total Assets	TAb	TAa	×	×	×	×
Shareholder's equity	SEb	SEa	×	×	×	×
Total liabilities and shareholder equity	TLSEb	TLSEa	×	×	×	×
Total income	×	×	TIb	TIa	×	×
Total revenues	×	×	TRb	TRa	×	×
Total expenses	×	×	TEb	TEa	×	×
Net income	×	×	NIb	NIa	×	×
Other changes in oper. Activity	×	×	×	×	COAb	COAa
Other changes in the Investing Act.	×	×	×	×	CIAb	CIAa
Other changes in the Financing Act.	×	×	×	×	CFAb	CFAa
Cash at End of Period.	×	×	×	×	CEPb	CEPa

Then, sum the abnormal returns over the event window to get the cumulative abnormal return (CAR).

Step 5: Statistical Testing.

Analyze the abnormal returns to determine if they are statistically significant. Calculate the mean and standard deviation of the abnormal returns within the event window. Perform statistical tests, such as a *t*-test and wiloxon, to assess whether the abnormal returns significantly differ from zero. This step helps confirm whether the event had a meaningful impact on the time series.

Significant CARs indicate that the event had a notable impact on the time series.

Step 6: Post-Event Analysis.

Optionally, extend our analysis beyond the immediate event window to assess the event's long-term impact. Update the ARIMA model parameters with post-event data if necessary. This step allows for a more comprehensive understanding of the event's effects over time, ensuring our analysis captures immediate and prolonged impacts.

This research employed the empirical event study methodology to assess the impact of the newly implemented 15 % VAT on various categories of non-financial companies in the Kingdom of Saudi Arabia. The investigation focused on non-financial Saudi-listed firms, analyzing two distinct periods: the second and third quarters of 2019 (preceding both the VAT implementation and COVID-19 outbreak) and the third and fourth quarters of 2020 (following the VAT introduction and amid the COVID-19 pandemic). The findings revealed that VAT increases affected businesses at different levels before and after the VAT imposition. To assess the impact of VAT, an 11-item index was employed, drawing from key financial statements. The Balance Sheet components included total assets, equity, and the liabilities-equity ratio. From the Income Statement, the index incorporated total income, total revenues, total expenses, and net income. The Cash Flow Statement contributed to changes in operational, investing, and financing activities and the cash balance at the end of the period. This comprehensive set of financial metrics was used to gauge the VAT's influence across various aspects of company performance.

**Fig. 1:**
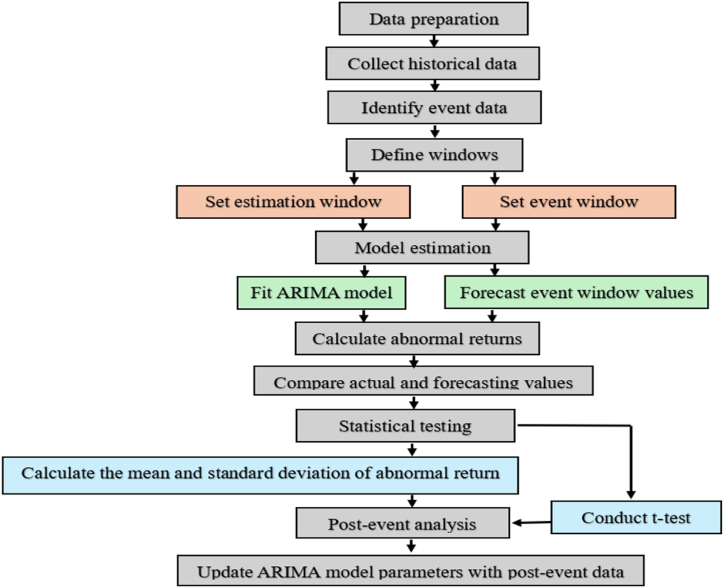
The process of integrating the Event Study Method (ESM) with the ARIMA model.

The flowchart of the proposed method is indicated in Fig. 1.

## 6 Results and discussion

Parameter adjustments in integrating event studies with ARIMA models are essential for enhancing the accuracy and reliability of the analysis. In an academic context, this involves a meticulous selection and fine-tuning of both ARIMA model parameters and event study parameters to suit the specific characteristics of the data and the event being studied. This study initially selects the ARIMA model parameters (p, d, q) based on the stock price data's autocorrelation and partial autocorrelation functions (ACF/PACF) in the pre-event window. Automated criteria like the Akaike Information Criterion (AIC) or Bayesian Information Criterion (BIC) further refine these parameters to minimize prediction errors [36]. The model that is adequate due to the residuals, the values of the parameters that ensure stationarity, reversibility, and the significance of the parameters is $ARIMA\;\left(1,0,0\right){\left(0,1,1\right)}_{11}$.

The pre-event window is set to 30 days, capturing sufficient data to model the underlying trend and volatility. Once the model is fitted, it predicts the expected stock prices during the 10-day event window around the earnings announcement. The stock prices observed during this period are then compared to these predictions to calculate abnormal returns. For instance, if the actual price on the first day of the event window is $105 and the predicted price is $100, the abnormal return is $5. Summing these abnormal returns over the event window provides the cumulative abnormal return (CAR), which is then tested for statistical significance. Adjusting parameters such as the length of the event window and the pre-event window and selecting appropriate statistical tests (e.g., parametric t-tests or non-parametric tests depending on the distribution of returns) are crucial steps. These adjustments ensure that the results accurately reflect the event's impact, accounting for the specific behavior of the time series data. An event window from 7 days before to 7 days after each event date (−7, +7) is employed. This range is chosen to align with the efficiency of stock markets. It is consistent with previous transportation and logistics research studies, which typically limit the event window to no more than 10 days [33]. Furthermore, selecting a shorter event window helps avoid overlapping event periods [34]. Moreover, a longer event window may diminish statistical power [35].

### 6.1 Variables

The variables selected for this study were meticulously chosen to comprehensively assess the financial impact of the VAT increase on non-financial listed companies in Saudi Arabia. These variables, derived from key financial statements such as the Balance Sheet, Income Statement, and Cash Flow Statement, were carefully selected to ensure a holistic evaluation of the companies' financial health and performance before and after the VAT hike. Total Assets (TA) and Shareholders' Equity (SE) were included to measure the size and ownership structure of the companies, providing insights into how their asset bases and equity positions were affected by the increased tax burden. Total Liabilities and Shareholders' Equity (TLSE) were selected to examine the companies' capital structure. This ensured that the balance sheet adhered to the fundamental accounting equation and reflected the overall financial stability in light of the VAT increase. From an income perspective, Total Income (TI), Total Revenues (TR), Total Expenses (TE), and Net Income (NI) were analyzed to understand the VAT's direct impact on revenue generation, cost management, and overall profitability. These variables are crucial for evaluating how the companies managed their core and ancillary income streams and whether the VAT increase led to higher operational costs, influencing their bottom line. Additionally, Cash Flow indicators such as Other Changes in Operating Activity (COA), Other Changes in Investing Activity (CIA), and Other Changes in Financing Activity (CFA) were included to capture changes in the companies' cash flow management strategies, reflecting how they adjusted their operations, investments, and financing activities in response to the increased tax burden. Cash at the End of the Period (CEP) was also examined to assess liquidity, which is critical for maintaining day-to-day operations and ensuring financial resilience. These carefully selected variables collectively offer a robust framework for analyzing the multifaceted effects of the VAT increase, providing critical insights into financial stability, operational efficiency, and overall economic performance within the non-financial sector of Saudi Arabia. By focusing on these comprehensive financial metrics, the study effectively captures the broad and nuanced impacts of the VAT policy, offering valuable empirical evidence for policymakers and stakeholders to consider when designing and implementing taxation strategies in similar economic contexts. These indicators offer critical insights into a company's financial stability, operational efficiency, and overall economic performance. The industry categorization employed in this study follows the classification system used by the Saudi Stock Exchange, also known as Tadawul. The analysis omitted financial companies from the sample, focusing solely on non-financial sectors.

Table 2, Table 3 provide descriptive statistics for each variable, comparing the periods before and after the VAT increase and the COVID-19 outbreak. The mean values reveal a significant decline in all key financial statement indicators following these events, supporting the study's hypothesis. The industry categories used in this analysis are based on the Saudi Stock Exchange (Tadawul) classification system, with financial companies excluded from the dataset.

**Table 2:** The descriptive statistical results for the key indicators of financial statements before the VAT increase and the onset of COVID-19. All numbers are presented in billions of riyals (10^9^).

Variables	Before Implementing VAT and the COVID-19 pandemic			
	Mean	Standard deviation	Min	Max
TAb	12.800	50.800	0.060	477.000
SEb	4.788	16.900	0.060	169.000
TLSEb	12.800	50.800	0.060	477.000
TIb	0.332	1.110	−0.003	8.3800
TRb	0.348	1.140	−0.105	8.700
TEb	0.234	0.980	−1.370	7.750
NIb	0.079	0.360	−0.720	2.680
COAb	0.055	0.970	−4.250	9.450
CIAb	0.191	1.080	−0.460	7.620
CFAb	−0.643	2.670	−20.30	0.520
CEPb	0.699	3.360	−0.005	37.40

Data source: Companies' annual reports author's calculation.

**Table 3:** The descriptive statistical results for the key indicators of financial statements following the VAT increase and the emergence of COVID-19. All numbers are presented in billions of riyals (10^9^).

Variables	After Implementing VAT and the COVID-19 pandemic			
	Mean	Standard deviation	Min	Max
TAa	12.800	51.700	0.043	503.000
SEa	4.650	16.600	0.014	165.000
TLSEa	12.800	51.700	0.043	503.000
TIa	0.320	1.210	−0.243	9.000
TRa	0.340	1.250	−0.178	9.000
TEa	0.230	0.996	−1.200	7.760
NIa	0.060	0.340	−0.610	2.770
COAa	0.090	0.600	−1.330	6.180
CIAa	0.016	0.270	−1.140	2.000
CFAa	−0.440	1.910	−15.700	0.000
CEPa	0.620	2.730	−0.034	30.00

Data source: Companies' annual reports author's calculation.

### 6.2 Industry comparison analysis

The analysis aims to assess the impact of VAT increases and the COVID-19 crisis on key financial indicators across various sectors, specifically in the energy sector, materials industry, capital goods sector, business and professional services, and transportation sector. The analysis aims to understand how these external shocks have influenced financial metrics such as Total Assets (TA), Shareholders’ Equity (SE), Total Liabilities and Shareholder Equity (TLSE), Total Income (TI), Total Revenues (TR), Total Expenses (TE), Net Income (NI), and other operational, investing, and financing activities. The rationale behind this analysis is crucial for several reasons. Firstly, it assesses the impact of VAT increases and the COVID-19 pandemic on financial metrics, providing insights into how these external economic pressures affect cash flow, revenue, and overall financial stability. The study compares financial data before and after these events and reveals how much such economic shocks influence vital indicators. Secondly, the analysis offers sector-specific insights, illustrating how industries respond to economic disturbances. For example, while the energy and materials industries experienced significant declines, the capital goods sector displayed varied impacts. Understanding these differences is essential for stakeholders to make informed decisions and develop effective strategies. Furthermore, the analysis identifies potential cash flow issues and revenue declines, critical for evaluating companies' short-term financial health and planning and risk management. It also examines operational, investing, and financing adjustments, shedding light on how companies adapt their strategies in response to economic pressures, including shifts in inventory levels, accounts receivable, and capital expenditures. Finally, by situating these findings within the broader economic context, such as global oil prices, inflation, and domestic demand fluctuations, the analysis helps to relate sector-specific challenges to overall economic conditions.

#### 6.2.1 Energy sector analysis

Table 4 presents a statistical analysis of various financial indicators before and after implementing a value-added tax (VAT) increase in Saudi Arabia. Before the VAT increase, the data shows that TA and TLSE had high mean values (24.34) with significant *t*-test results (3.8692 for TA and 15.1238 for TLSE), indicating aggregate solid value and compliance with the accounting equation. The SE mean was 5.34, showing a relatively lower residual interest post-liabilities. TI and TR had mean values close to each other, with TI at 0.95 and TR at 1.03, though only TI showed statistical significance in the *t*-test. NI's mean value was 0.57, indicating modest profitability. Adjustments like COA and CFA showed negative mean values, suggesting decreased operating and financing activities, respectively. The significance of COA's *t*-test (−0.0762) and Wilcoxon test results for COA and CFA highlights notable changes in these activities.

**Table 4:** Cumulative abnormal returns in the Energy industry during different event window periods.

Scenario	Variables	Window	Statistical analysis			
			Mean	Std. dev.	*t*-test	Wilcoxon
Before the rising value-added tax, 2019 (VAT = 5 %)	TA	[-7,+7]	24.3400	33.7800	3.8692	2.4913∗∗
	SE	[-7,+7]	5.3400	5.4600	2.2455	1.7104
	TLSE	[-7,+7]	24.3400	33.7800	15.1238	8.6791
	TI	[-7,+7]	0.9500	1.2300	0.2962∗∗∗	0.1212
	TR	[-7,+7]	1.0300	1.2900	0.2292	0.5615
	TE	[-7,+7]	0.4500	0.5700	0.0597	0.0589
	NI	[-7,+7]	0.5700	0.7500	0.2485	0.2745
	COA	[-7,+7]	−0.2300	0.3900	−0.0762∗	−0.2232∗
	CIA	[-7,+7]	0.2800	0.8700	0.2781	0.1217
	CFA	[-7,+7]	−0.2500	0.4600	−0.2084	−0.1341∗∗
	CEP	[-7,+7]	0.8900	0.9900	0.8758	0.6249
After the rising value-added tax, 2020 (VAT = 15 %)	TA	[-7,+7]	22.9700	32.1300	3.1489	1.6710
	SE	[-7,+7]	3.9700	4.0200	0.3563	0.2177
	TLSE	[-7,+7]	22.9700	32.9900	12.7117	11.5261
	TI	[-7,+7]	0.6100	0.9100	0.0791∗∗∗	0.0995
	TR	[-7,+7]	0.6300	1.1300	0.1488	0.1292∗∗
	TE	[-7,+7]	0.6000	0.6300	0.4537	0.1347
	NI	[-7,+7]	0.0110	0.5200	0.0571	0.3590
	COA	[-7,+7]	0.1100	−0.2400	0.0111∗	0.0818
	CIA	[-7,+7]	−0.00068	−0.0012	−0.0065	−0.0032∗∗
	CFA	[-7,+7]	−0.5700	0.8400	−0.4987	−1.9266
	CEP	[-7,+7]	0.5000	0.8000	0.3650	1.8250

∗∗∗, ∗∗, and ∗ represent 1, 5, and 10 % significance levels, respectively.

Post-VAT increase shows a noticeable decrease in TA (mean of 22.97) and SE (mean of 3.97), reflecting a potential impact of the higher tax rate on the company's resources and shareholders' stakes. TI and TR also decreased (mean TI at 0.61 and mean TR at 0.63), with TR showing statistical significance in the Wilcoxon test, indicating a potential drop in earnings from core activities. NI dropped significantly to 0.011, suggesting a sharp decline in profitability. COA and CFA exhibited positive mean values, with COA showing statistical significance, indicating changes in operating and financing activities post-VAT increase. CEP decreased to a mean of 0.5, showing reduced liquidity at the end of the period.

Moreover, the comparative analysis indicates that the VAT increase significantly impacted the financial health of companies in the Energy industry. Total Assets and Shareholders' Equity showed reductions, reflecting constrained resources and reduced stakeholder interest. Revenue-related indicators like TI and TR also declined, impacting profitability as evidenced by the drop in NI. The shifts in COA and CFA suggest changes in operating and financing strategies post-VAT increase. The overall decrease in CEP highlights reduced cash reserves, indicating liquidity challenges following the tax hike.

#### 6.2.2 Materials industry analysis

The analysis of key financial indicators before and after the rise in value-added tax (VAT) from 5 % to 15 % reveals notable shifts in the materials industry's economic health, as shown in Table 5. Before the VAT increase in 2019, Total Assets (TA) and Total Liabilities and Shareholders' Equity (TLSE) had a mean of 14.280 with high standard deviations, indicating considerable variability in asset values and balance sheet compositions across companies. Shareholders' Equity (SE) was relatively low but statistically significant, with a *t*-test value of 3.0151, suggesting a stable yet modest equity base among the firms. Total Revenues (TR) and Total Expenses (TE) also showed significant variations, with TR showing a mean of 0.210 and TE at 0.190, indicating a closely matched revenue-expense relationship. However, both values are relatively low, reflecting constrained profitability.

**Table 5:** Cumulative abnormal returns in the materials industry during different event window periods.

Scenario	Variables	Window	Statistical analysis			
			Mean	Std. dev.	*t*-test	Wilcoxon
Before the rising value-added tax, 2019 (VAT = 5 %)	TA	[-7,+7]	14.280	47.040	2.2701	1.4616
	SE	[-7,+7]	7.170	24.770	3.0151∗∗	2.2965
	TLSE	[-7,+7]	14.280	47.040	8.8729	5.0919
	TI	[-7,+7]	0.200	0.670	0.0623	0.0255
	TR	[-7,+7]	0.210	0.650	0.0467	0.1144∗∗
	TE	[-7,+7]	0.190	0.740	0.0252	0.0249∗∗
	NI	[-7,+7]	0.0087	0.140	0.0037	0.0042
	COA	[-7,+7]	0.041	0.500	0.0135	0.0397
	CIA	[-7,+7]	0.390	1.410	0.3874	0.1695
	CFA	[-7,+7]	−0.920	3.170	−0.7669∗∗∗	−0.4936∗∗∗
	CEP	[-7,+7]	1.310	5.500	1.2892	0.9199
After the rising value-added tax, 2020 (VAT = 15 %)	TA	[-7,+7]	13.490	44.080	1.8493∗∗∗	0.9813
	SE	[-7,+7]	6.930	24.260	0.6219	0.3800∗
	TLSE	[-7,+7]	13.490	44.080	7.4654	6.7692
	TI	[-7,+7]	0.240	0.950	0.0311∗	0.0392∗∗
	TR	[-7,+7]	0.270	1.060	0.0637	0.0554
	TE	[-7,+7]	0.170	0.840	0.1285	0.0382
	NI	[-7,+7]	0.058	0.170	0.0300∗∗∗	0.01892
	COA	[-7,+7]	0.026	0.250	0.0260	0.0193∗∗∗
	CIA	[-7,+7]	−0.039	0.300	−0.0374	−0.0186
	CFA	[-7,+7]	−0.610	2.410	−0.5337	−2.0618
	CEP	[-7,+7]	1.070	4.430	0.7811	3.9055

∗∗∗, ∗∗, ∗ represent 1, 5, and 10 % significance levels, respectively.

After the VAT hike in 2020, the data presents a different landscape. Total Assets (TA) slightly decreased to 13.490, showing a mild impact on the assets despite the higher tax burden. Shareholders’ Equity (SE) also dipped to 6.930 but lacked significant *t*-test values, indicating no substantial shift in equity holdings. Interestingly, Total Income (TI) and Net Income (NI) showed an increase in means (0.240 and 0.058, respectively) and were significant at different levels, indicating better income management and profitability despite higher operating costs. Other Changes in Operating Activity (COA) and Other Changes in Investing Activity (CIA) did not display significant alterations, suggesting consistent operational and investment adjustments without drastic changes due to the VAT increase.

Comparatively, the financial health and performance of companies post-VAT rise demonstrate resilience. Despite an initial strain on total assets and shareholders’ equity, the total income and net income improvement reflect adaptive strategies that enhance profitability. The significant drop in Cash at the End of the Period (CEP) from 1.310 to 1.070, alongside the decrease in Other Changes in Financing Activity (CFA), indicates a tighter liquidity position and potentially reduced external financing. Overall, the companies managed to sustain their financial stability and operational efficiency despite the heightened tax environment, highlighting their adaptability and robust financial management practices.

#### 6.2.3 Capital goods industry analysis

The comparative analysis of the data presented in Table 6 focuses on the periods before and after the rise in value-added tax (VAT) in Saudi Arabia's capital goods industry. Before the VAT increase in 2019, when the rate was 5 %, Total Liabilities and Shareholder Equity (TLSE) showed a mean of 1.420 with a t-statistic of 1.5906, significant at the 5 % level. Total Income (TI) had a mean of 0.036 with a t-statistic of 0.0045, which was also significant at 5 %. Other Changes in Operating Activity (COA) showed a mean of 0.015 and was significant at the 1 % level in the Wilcoxon test with a value of 0.0138. Cash at the End of Period (CEP) had a mean of 0.041 and was significant at the 10 % level in the Wilcoxon test with a value of 0.2207.

**Table 6:** Cumulative abnormal returns in the capital goods industry during different event window periods.

Scenario	Variables	Window	Statistical analysis			
			Mean	Std. dev.	*t*-test	Wilcoxon
Before the rising value-added tax, 2019 (VAT = 5 %)	TA	[-7,+7]	1.420	1.030	1.4376	0.860838
	SE	[-7,+7]	0.510	0.380	0.3244	−0.21294
	TLSE	[-7,+7]	1.420	1.030	1.5906∗∗	−0.80323
	TI	[-7,+7]	0.036	0.046	0.0045∗∗	0.004831∗∗
	TR	[-7,+7]	0.038	0.044	0.0180	0.013875
	TE	[-7,+7]	0.013	0.052	0.0132	0.008664
	NI	[-7,+7]	−0.00014	0.015	−0.00036	−0.0041
	COA	[-7,+7]	0.015	0.390	0.0081	0.0138∗∗∗
	CIA	[-7,+7]	−0.017	0.053	−0.0394	−0.0463
	CFA	[-7,+7]	−0.017	0.026	−0.0472	−0.0389
	CEP	[-7,+7]	0.041	0.050	0.0829	0.2207∗
After the rising value-added tax, 2020 (VAT = 15 %)	TA	[-7,+7]	1.440	0.960	0.7894	1.095
	SE	[-7,+7]	0.530	0.410	0.4241	−0.1444
	TLSE	[-7,+7]	1.440	0.960	0.8090∗∗	0.5864∗
	TI	[-7,+7]	0.035	0.053	0.0195∗∗	0.0224∗
	TR	[-7,+7]	0.046	0.055	0.0407	0.0326
	TE	[-7,+7]	0.013	0.054	0.01658	0.0122
	NI	[-7,+7]	0.0065	0.025	0.0269	0.0239
	COA	[-7,+7]	0.0025	0.043	0.0054∗∗∗	−0.0880
	CIA	[-7,+7]	0.0048	0.0088	0.0033	0.0614
	CFA	[-7,+7]	−0.010	0.024	−0.0174	−0.0203∗∗∗
	CEP	[-7,+7]	0.044	0.044	0.0571	0.0543

∗∗∗, ∗∗, ∗ represent 1, 5, and 10 % significance levels, respectively.

After the VAT increase to 15 % in 2020, TLSE showed a slightly higher mean of 1.440 but a lower t-statistic of 0.8090, still significant at 5 %. It also became substantial in the Wilcoxon test at 10 % with a value of 0.5864. TI maintained a similar mean at 0.035 but showed a higher t-statistic of 0.0195, significant at 5 %, and became substantial in the Wilcoxon test at 10 % with a value of 0.0224. COA's mean decreased to 0.0025 and lost its significance in the Wilcoxon test. A new significant effect emerged for Other Changes in Financing Activity (CFA), showing a negative mean of −0.010 and becoming significant at 1 % in the Wilcoxon test with a value of −0.0203.

Comparing the two periods, we see that while TLSE and TI remained significantly positive, their patterns changed. TLSE's effect seemed to weaken slightly in the *t*-test but gained significance in the Wilcoxon test. TI's effect strengthened overall, becoming significant in both tests. The loss of importance for COA and the emergence of a substantial adverse impact for CFA after the VAT increase are notable changes, potentially indicating shifts in companies' operational and financing activities in response to the higher tax rate.

#### 6.2.4 Business and professional services industry analysis

Table 7 shows significant changes in various financial variables following the Value Added Tax (VAT) increase from 5 % to 15 % between 2019 and 2020. Several financial indicators showed significant effects before the VAT increase 2019 (VAT at 5 %). Total Assets (TA) and Shareholders' Equity (SE) demonstrated substantial positive impacts at the 5 % level, with t-statistics of 1.8426 and 0.5215, respectively. Total Income (TI) showed a positive mean of 0.120 and was significant at the 1 % level in the Wilcoxon test (0.0161). Net Income (NI) also showed a significant positive effect at the 1 % level in the *t*-test (0.0182). Other Changes in Investing Activity (CIA) were significant at the 5 % level in the Wilcoxon test (0.1498). These results suggest that before the VAT increase, companies in this industry were experiencing growth in assets, equity, and income, with positive changes in investing activities. After the VAT increase to 15 % in 2020, we observed a shift in the pattern of significant effects. SE remained substantial but is now at the 1 % level with a t-statistic of 0.5601. Total Revenues (TR) became significant at the 5 % level in both the *t*-test (0.02303) and the Wilcoxon test (0.0184), which wasn't the case before the VAT increase. Interestingly, Other Changes in Operating Activity (COA) showed a significant adverse effect at the 10 % level in the Wilcoxon test (−0.0494), and Other Changes in Financing Activity (CFA) became significant at the 5 % level in the Wilcoxon test (−0.2433), both of which were not significant before the VAT increase.

**Table 7:** Cumulative abnormal returns in the business and professional services industry during different event window periods.

Scenario	Variables	Window	Statistical analysis			
			Mean	Std. dev.	*t*-test	Wilcoxon
Before the rising value-added tax, 2019 (VAT = 5 %)	TA	[-7,+7]	1.820	0.700	1.8426∗∗	1.1033
	SE	[-7,+7]	0.820	0.400	0.5215∗∗	−0.3424
	TLSE	[-7,+7]	1.820	0.700	2.0387	−1.0295
	TI	[-7,+7]	0.120	0.089	0.0150	0.0161∗∗∗
	TR	[-7,+7]	0.120	0.088	0.0570	0.0438
	TE	[-7,+7]	0.031	0.050	0.0314	0.0206
	NI	[-7,+7]	0.071	0.066	0.0182∗∗∗	0.0208
	COA	[-7,+7]	0.059	0.082	0.0318	0.0545
	CIA	[-7,+7]	0.055	0.091	0.1274	0.1498∗∗
	CFA	[-7,+7]	−0.330	0.280	−0.9165	−0.7558
	CEP	[-7,+7]	0.110	0.107	0.2225	0.5923
After the rising value-added tax, 2020 (VAT = 15 %)	TA	[-7,+7]	1.680	0.700	0.9205	1.2775
	SE	[-7,+7]	0.700	0.200	0.5601∗∗∗	0.4360
	TLSE	[-7,+7]	1.680	0.700	0.9438	0.6842
	TI	[-7,+7]	0.024	0.044	0.0134	0.0153
	TR	[-7,+7]	0.026	0.046	0.02303∗∗	0.0184∗∗
	TE	[-7,+7]	−0.017	0.055	−0.0216	−0.0159
	NI	[-7,+7]	−0.018	0.075	−0.0745	−0.0662
	COA	[-7,+7]	0.014	0.075	0.0302	−0.0494∗
	CIA	[-7,+7]	−0.0088	0.019	−0.0616	−0.0112
	CFA	[-7,+7]	−0.120	0.350	−0.2097	−0.2433∗∗
	CEP	[-7,+7]	0.130	0.099	0.1687	0.16039

∗∗∗, ∗∗, ∗ represent 1, 5, and 10 % significance levels, respectively.

Comparing the two periods, the VAT increase appears to have had a mixed impact on the business and professional services industry. While SE remained positively affected, TA lost its significance. The emergence of significant effects on TR suggests that companies might focus more on revenue generation post-VAT increase. However, the new adverse significant effects on COA and CFA indicate potential challenges in operating and financing activities. It's also noteworthy that NI and CIA lost their positive significant effects after the VAT increase, which could suggest that companies are facing difficulties in maintaining profitability and positive investing activities under the higher tax rate. These changes paint a picture of an industry adapting to a new tax environment, with some positive developments in revenue but potential challenges in other areas of financial performance.

#### 6.2.5 Transportation sector analysis

The results in Table 8 reveal significant changes in the financial landscape of the Transportation Sector following the VAT increase from 5 % in 2019 to 15 % in 2020. Before the VAT increase in 2019, Total Assets (TA) and Total Liabilities and Shareholders' Equity (TLSE) showed a mean of 2.460 with a standard deviation of 1.500. TA had a significant *t*-test value of 2.4906, while TLSE's *t*-test value was 2.7556. Total Income (TI) had a small but highly significant mean of 0.160 (*t*-test: 0.0201, p < 0.01). Current Operating Assets (COA) showed a mean of 0.140 with a substantial *t*-test value of 0.0755 (p < 0.05). Notably, Current Financial Assets (CFA) had a negative mean of −0.107 and a significant Wilcoxon test value of −0.2451 (p < 0.05). After the VAT increase in 2020, we observed some changes. TA and TLSE increased to a mean of 2.990 with a higher standard deviation of 1.840, but neither showed statistical significance. Total Expenses (TE) increased from 0.095 to 0.105 and became statistically significant (*t*-test: 0.1339, p < 0.05; Wilcoxon: 0.0983, p < 0.1). Current Investing Assets (CIA) saw a substantial increase from 0.0025 to 0.091 and became statistically significant (*t*-test: 0.0637, p < 0.05). CFA remained negative at −0.038 and maintained statistical significance (Wilcoxon: 0.0771, p < 0.01).

**Table 8:** Cumulative abnormal returns in the Transportation Sector during different event window periods.

Scenario	Variables	Window	Statistical analysis			
			Mean	Std. dev.	*t*-test	Wilcoxon
Before the rising value-added tax, 2019 (VAT = 5 %)	TA	[-7,+7]	2.460	1.500	2.4906	1.4913∗
	SE	[-7,+7]	1.360	0.880	0.8651	−0.5678
	TLSE	[-7,+7]	2.460	1.500	2.7556	−1.3915
	TI	[-7,+7]	0.160	0.059	0.0201∗∗∗	0.0214
	TR	[-7,+7]	0.150	0.090	0.0713	0.0547
	TE	[-7,+7]	0.095	0.048	0.0964	0.0633
	NI	[-7,+7]	0.051	0.050	0.0131	0.0151
	COA	[-7,+7]	0.140	0.210	0.0755∗∗	0.1293
	CIA	[-7,+7]	0.0025	0.053	0.0058	0.0068
	CFA	[-7,+7]	−0.107	0.130	−0.2972	−0.2451∗∗
	CEP	[-7,+7]	0.270	0.240	0.5462	1.4538
After the rising value-added tax, 2020 (VAT = 15 %)	TA	[-7,+7]	2.990	1.840	1.6382	2.2736
	SE	[-7,+7]	1.270	0.770	1.0161	1.1196
	TLSE	[-7,+7]	2.990	1.840	1.6798	1.2177
	TI	[-7,+7]	0.100	0.075	0.0559	0.0640∗∗
	TR	[-7,+7]	0.083	0.072	0.0735	0.0589
	TE	[-7,+7]	0.105	0.048	0.1339∗∗	0.0983∗
	NI	[-7,+7]	−0.025	0.070	−0.0103	−0.0092
	COA	[-7,+7]	0.148	0.280	0.0319	−0.0522
	CIA	[-7,+7]	0.091	0.210	0.0637∗∗	0.1165
	CFA	[-7,+7]	−0.038	0.035	−0.0664	−0.0771∗∗∗
	CEP	[-7,+7]	0.310	0.280	0.4023	0.3824

∗∗∗, ∗∗, ∗ represent 1, 5, and 10 % significance levels, respectively.

Comparing the two periods, we see that while TA and TLSE grew (by 21.5 %), they lost statistical significance, suggesting increased variability in the sector. The increase in TE (10.5 %) likely reflects the impact of the higher VAT. The dramatic rise in CIA (from 0.0025 to 0.091, a 3540 % increase) coupled with the continued negative CFA suggests a shift in asset allocation strategies. TI decreased from 0.160 to 0.100 and lost statistical significance, while Net Income (NI) shifted from a positive 0.051 to a negative −0.025, though neither NI value was statistically significant. These changes indicate that while the sector continued to grow in assets, it faced increased expenses and potential profitability challenges following the VAT increase.

#### 6.2.6 Comparison and overall finding

Table 9 presents a comparison of the average effect across all industries. The financial indicators across the Energy, Materials, Capital Goods, Business and Professional Services, and Transportation sectors reveal diverse performance patterns and economic dynamics. The Business and Professional Services sector emerges as a standout performer, showing robust growth in total assets (7.53 %), total liabilities and stockholders' equity (7.52 %), total income (75.28 %), and total revenue (74.34 %). This sector's expansion is further evidenced by significant increases in expenses (144.92 %) and net income (117.63 %), suggesting a period of intense business activity and successful growth strategies. In contrast, the Transportation sector presents a more complex picture. Despite experiencing a substantial decline in total assets and liabilities (−19.76 %), it managed to achieve considerable growth in total income (34.82 %), revenue (42.35 %), and net income (140.23 %). This paradoxical performance might indicate a successful restructuring or a shift towards asset-light business models within the industry. The sector's mixed cash flow results, with a decline in operating activities (−6.58 %) but substantial growth in investing activities (3351.04 %) and financing activities (60.22 %), further support this interpretation.

**Table 9:** Comparative analysis of key financial indicators across industries before and after VAT increase and COVID-19 onset.

Variables	Various sectors (%)				
	Energy (%)	Materials (%)	Capital goods (%)	Business and Professional Services (%)	Transportation (%)
TA	5.64	5.64	−0.94	7.53	−19.76
SE	24.46	2.82	−3.76	13.17	6.58
TLSE	5.64	5.64	−0.94	7.52	−19.76
TI	33.87	−16.93	1.88	75.28	34.82
TR	36.70	−25.41	−19.76	74.34	42.35
TE	−31.99	12.24	0.00	144.92	−10.35
NI	92.22	−533.57	4578.16	117.63	140.23
COA	140.21	34.82	79.05	71.52	−6.58
CIA	94.10	103.51	121.39	109.16	−3351.04
CFA	−123.27	32.00	36.70	59.28	60.22
CEP	41.41	16.93	−7.53	−14.12	−14.11

The Energy sector demonstrates robust financial health across multiple indicators. It leads in stockholders' equity growth (24.46 %) and shows solid increases in total income (33.87 %) and revenue (36.70 %). The sector's significant growth in cash from operating activities (140.21 %), coupled with high capital expenditure growth (41.41 %), suggests a period of expansion and investment in long-term assets. However, the decline in cash from financing activities (−123.27 %) might indicate a reduced reliance on external funding sources. Capital Goods and Materials sectors present more challenging scenarios. While the Capital Goods sector experienced an extraordinary increase in net income (4578.16 %) despite no change in total expenses, it faced declines in total assets (−0.94 %), stockholders' equity (−3.76 %), and revenue (−19.76 %). This unusual pattern might be attributed to significant non-operational gains or one-time events. The Materials sector, on the other hand, saw moderate growth in assets (5.64 %) and equity (2.82 %) but faced substantial declines in revenue (−25.41 %) and net income (−533.57 %), hinting at potential market challenges or pricing pressures. The data reveals varying approaches to capital expenditure across all sectors. While Energy (41.41 %) and Materials (16.93 %) increased their investments in long-term assets, the other sectors reduced their capital expenditures: Capital Goods (−7.53 %), Business and Professional Services (−14.12 %), and Transportation (−14.11 %). This divergence might reflect differing responses to economic uncertainties or shifts in investment strategies across industries.

The analysis shows a dynamic economic landscape where sectors respond differently to market conditions. Business and Professional Services and Energy are in expansionary phases, while Transportation is navigating significant structural changes. Capital Goods and Materials face more challenging environments, necessitating strategic adjustments. These varying sectoral performances underscore the complexity of the current economic climate and the importance of sector-specific strategies in navigating financial success.

Table 10 presents a comparative analysis of key financial indicators across 16 industry sectors, examining 11 financial variables as percentages relative to the average across all industries. The variables include metrics like Total Assets, Shareholders' Equity, Total Income, Net Income, and various cash flow measures. Positive percentages indicate an increase compared to the industry average, while negative percentages show a decrease. Some sectors exhibit extreme variations in specific indicators, such as Capital goods showing a 4578.16 % increase in Net Income or Consumer services with an 8087.30 % increase in Cash from Investing Activities. Other sectors consistently perform above or below average across multiple indicators. For instance, the Energy sector shows positive growth in most indicators except Total Expenses and Cash from Financing Activities. In contrast, the healthcare equipment and services sector shows negative growth across all indicators.

**Table 10:** Aggregate changes in average values of key financial indicators across all industries.

Sectors	Variables (%)										
	TA	SE	TLSE	TI	TR	TE	NI	COA	CIA	CFA	CEP
Energy	5.64	24.46	5.64	33.87	36.7	−31.99	92.22	140.21	94.1	−123.27	41.41
Materials	5.64	2.82	5.64	−16.93	−25.41	12.24	−533.57	34.82	103.51	32	16.93
Capital goods	−0.94	−3.76	−0.94	1.88	−19.76	0	4578.16	79.05	121.39	36.7	−7.53
Commercial and professional Svc.	7.53	13.17	7.52	75.28	74.34	144.92	117.63	71.52	109.16	59.28	−14.12
Transportation	−19.76	6.58	−19.76	34.82	42.35	−10.35	140.23	−6.58	−3351.04	60.22	−14.11
Consumer durables and apparel	9.41	8.46	9.41	29.17	4.70	−12.23	232.43	−94.10	−625.79	164.68	−51.75
Consumer services	2.82	−6.58	2.82	48.93	48.93	24.46	143.03	877.05	8087.30	−466.75	−6.58
Media and entertainment	7.52	−29.17	7.52	−5.64	0.94	10.35	12.23	−178.79	59.28	6176.00	33.87
Retailing	3.76	16.93	3.76	60.22	55.52	10.35	123.27	−27.29	−158.09	8.46	−25.41
Food and staples retailing	0	6.58	0	15.99	15.99	5.64	51.75	−13.17	−1081.25	−2.82	−56.46
Food and beverages	−1.88	−7.52	−1.88	−8.46	−10.35	1.88	−103.51	87.51	1091.60	42.34	2.82
Health care equipment and Scv.	−8.46	−7.52	−8.46	−35.75	−34.81	−21.64	−50.81	−113.86	−76.22	−27.29	−13.17
Pharma, biotech and life science	6.58	15.05	6.58	−14.11	−24.46	−5.64	−101.63	118.57	62.11	55.52	8.46
Telecommunication services	0	−2.82	0	−3.76	−2.82	0.94	−21.64	172.21	68.69	42.34	31.99
Utilities	−4.70	4.70	−4.70	15.99	12.23	10.35	17.87	32.93	186.32	25.41	−53.63
Real estate Mgmt and Dev't	−4.70	2.82	−4.70	54.58	52.69	−5.64	264.43	92.22	200.44	−587.20	−18.82

**Fig. 2:**
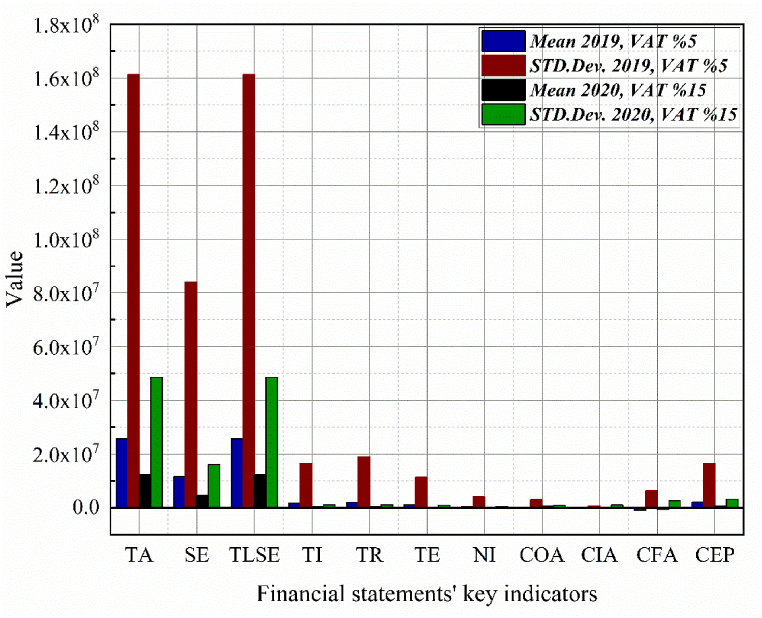
Changes in the average values of financial variables across all industries.

Fig. 2 illustrates the effects of the value-added tax (VAT) increase from 5 % in 2019 to 15 % in 2020 on key financial statement indicators. It compares these indicators, showing average values and standard deviations across the sampled companies. Total Total Assets (TA), Total Assets (TA), Total Liabilities and Shareholders' Equity (TLSE) both substantially declined, with mean values dropping from approximately 25.58 million to 12.31 million. This decrease indicates a considerable reduction in the aggregate value of companies' resources and overall financial stability, highlighting the adverse effects of the higher VAT rate on business assets and equity structures.

Shareholders' Equity (SE) experienced a notable decrease, with mean values falling from 11.55 million to approximately 4.57 million. This reduction underscores companies' challenges in maintaining profitability and financial resilience amid increased tax burdens. Total Income (TI) and Total Revenues (TR) also significantly decreased, with mean values dropping from approximately 1.73 million to 316,234 and from 1.95 million to 331,605, respectively. These declines suggest a substantial impact on companies' revenue-generating capabilities, likely due to reduced consumer spending and increased operational costs resulting from the higher VAT. Net Income (NI) and Total Expenses (TE) further illustrate the financial strain imposed by the VAT increase. Net Income mean values dropped from approximately 406,109 to 74,624, indicating a sharp decline in overall profitability. Similarly, Total Expenses decreased from about 1.18 million to 223,495, reflecting significant cost-cutting measures companies likely implemented to cope with the reduced income. Other financial activities also showed notable fluctuations; Other Changes in Operating Activity (COA) shifted from a negative mean of approximately −168,936 to a positive 523,520, indicating changes in operational adjustments. Meanwhile, Cash at the End of the Period (CEP) mean values fell from around 1.99 million to 666,663, emphasizing the impact on liquidity and the available cash reserves necessary for sustaining operations. These changes across financial indicators highlight businesses' broader economic and financial challenges after the VAT increase.

Ultimately, the industry comparison analysis was distilled to highlight the most critical findings across five key sectors: Energy, Materials, Capital Goods, Business and Professional Services, and Transportation. These sectors were selected based on their significant role in the Saudi economy and their diverse responses to the Value Added Tax (VAT) increase. The analysis revealed that the Energy sector exhibited robust growth in Total Revenue and Shareholders' Equity, showcasing its resilience and ability to adapt to fiscal changes. In contrast, the Materials and Capital Goods sectors encountered considerable challenges, with marked Net Income and Total Revenue declining, indicating their susceptibility to increased taxation. The Transportation sector displayed strategic shifts in asset allocation despite a decline in overall assets, suggesting an adaptive response to the new tax environment. The Business and Professional Services sector also showed strong performance, particularly in revenue and net income, highlighting its flexibility in managing the tax changes. The decision to focus on these five sectors, out of the original 16, is rooted in their economic significance and ability to represent the broader non-financial market in Saudi Arabia. These sectors were chosen for their substantial economic contribution and diverse industry characteristics. By concentrating on these sectors, the study provides a more detailed and focused analysis of the VAT's impact, allowing for a deeper understanding of the tax's effects on different industries. This selective focus ensures that the study maintains depth and specificity, avoiding the dilution of insights that might occur if all 16 sectors were analyzed. Moreover, the selected sectors offer a balanced view of how various industries, from capital-intensive sectors like Energy and Materials to service-oriented sectors like Business and Professional Services, respond to indirect taxation. This approach enables the study to draw more precise conclusions and provide targeted recommendations relevant to policymakers and industry stakeholders. By focusing on these critical sectors, the analysis delivers actionable insights vital for understanding the broader implications of VAT on the Saudi economy and guiding strategic planning within these industries.

### 6.3 Sectoral responses to VAT increases amid gross domestic product (GDP) changes

The analysis of financial indicators across different sectors in Saudi Arabia following the VAT increase and the onset of the COVID-19 pandemic reveals significant variations in how these sectors responded to fiscal and economic pressures. The Energy sector, for example, exhibited strong growth in shareholders' equity (24.46 %) and revenue (36.7 %), demonstrating its resilience during a period of economic uncertainty. This sector's robust performance can be largely attributed to the partial recovery of oil prices in the later stages of the period, which helped offset the initial impact of the VAT increase and the economic slowdown. Despite the challenges posed by a shrinking GDP, particularly in 2020, when Saudi Arabia's GDP contracted by 4.1 %, the Energy sector's strong linkage to global oil markets allowed it to maintain and even enhance its financial stability [28]. In contrast, the Materials and Capital Goods sectors faced more pronounced difficulties, reflected in significant revenue and net income declines. The Materials sector, in particular, saw a staggering 533.57 % decrease in net income, highlighting the severe impact of reduced domestic and international demand. These struggles can be directly linked to the broader economic environment, where GDP contraction affected sectors heavily dependent on industrial activity and construction. The reduction in domestic consumption, compounded by the VAT increase and global trade disruptions, further exacerbated the financial strain on these sectors. The GDP data, showing a 6.7 % contraction in the oil industry and a 2.3 % decline in non-oil industries, underscores these sectors' challenges during this economic downturn. On the other hand, the Business and Professional Services sector demonstrated remarkable resilience, with significant increases in total income (75.28 %) and net income (117.63 %). This sector's ability to thrive in a contracting economy suggests effective adaptation strategies, such as cost optimization, digital transformation, and diversified service offerings that align with the changing economic landscape. The sector's strong performance is particularly noteworthy given the broader context of a declining GDP, which typically exerts downward pressure on business services. However, the sector's focus on innovation and efficiency appears to have mitigated the negative impacts of the economic slowdown, allowing it to capitalize on the relatively stable non-oil GDP components. These findings highlight the critical importance of considering macroeconomic factors, such as GDP growth or contraction, when evaluating the impact of fiscal policies like VAT increases. While sectors like Energy and Business and Professional Services have shown resilience and adaptability, others like Materials and Capital Goods have struggled under the dual pressures of a higher tax burden and a contracting economy. The significant variability in sectoral performance suggests that a one-size-fits-all policy approach may not be effective. Policymakers should consider implementing targeted support measures for vulnerable sectors, particularly those most affected by GDP fluctuations and global economic uncertainties. Future research should integrate GDP trends and other macroeconomic indicators into analyses of fiscal policy impacts to provide a more nuanced understanding of how these policies interact with the broader economic environment in emerging markets like Saudi Arabia.

### 6.4 Discussions on critical perspectives

In this section, statistical results are interpreted through an in-depth analytical discussion to elucidate the significance behind the data; by addressing the fundamental question, "So what?" a contextual understanding is provided to enhance reader comprehension. Empirical findings are meticulously demonstrated and analyzed, offering a robust comparison with previous studies to highlight consistencies or deviations. This critical evaluation is performed using a comprehensive extended analysis, ensuring a thorough examination of the data. Furthermore, improvements are made to simplify complex concepts, making the findings more accessible and easier for a broader audience to understand. The results are presented and meaningfully interpreted through this approach, underscoring their relevance and implications in the field.

A severe increase in VAT has complex and multifaceted impacts on inter-industry volatility in corporate financial reporting metrics. While some industries may experience heightened costs, potentially leading to reduced profit margins and increased consumer prices, others may benefit from the government's enhanced revenue, which could be redirected towards public investment or subsidies. The dynamic effects of VAT adjustments necessitate a nuanced examination, particularly when considering the contrasting outcomes of VAT decreases. In sectors where VAT is reduced, consumer prices often drop, improving living standards and potentially leading to higher disposable incomes and increased tax revenues from higher taxable incomes. This dynamic underscores the importance of understanding sector-specific responses to tax policy changes. Historical examples, such as the United States corporate tax cuts in 2017, demonstrate how targeted tax reductions can spur economic growth, boost corporate investments, and increase job creation, ultimately contributing to more robust financial performance. These observations highlight the critical role of strategic tax policy in managing economic stability and growth.

Reducing the VAT rate directly lowers the prices of goods and services, as VAT is added to the cost of products. This price decrease boosts consumer purchasing power, increasing spending on a broader range of goods and services. Consequently, businesses experience higher sales volumes, potentially resulting in more significant revenue and profits. The surge in consumer spending enhances domestic demand, driving economic growth and prompting firms to expand. Despite a lower VAT rate, overall tax revenues may rise due to increased sales and income tax from higher employment and thriving businesses. Furthermore, improved public services and infrastructure from increased government revenues can enhance the quality of life. This cumulative effect of increased consumer spending, higher business sales, and improved public services fosters overall economic growth, supporting the recommendation for nations to consider reducing VAT rates to stimulate prosperity and financial stability. Contrariwise, an increase in VAT can impact various sectors differently, with potential consequences for businesses and economic growth. When businesses cannot pass the total VAT increase onto consumers, they encounter higher capital costs due to advanced input VAT, which is paid on production inputs. This situation can alter demand for products and influence business performance and costs. Although VAT is designed to be neutral and not distort business decisions, its neutrality may be compromised if not all elements are considered. Ormaechea et al. [38] highlight that VAT design features, such as exemptions and differential tax rates, can lead to inefficient resource allocation and growth issues. Exemptions might result in tax distortions affecting business decisions, while differential taxes and net VAT structures further complicate the economic landscape. Research by Cnossen [39] indicates that these design elements can distort business entry and resource distribution, potentially impeding economic growth.

Furthermore, differentiated VAT rates, which apply different tax rates to various goods and services, can create inefficiencies in the market. Lower tax rates on certain items distort prices and consumer choices, complicating business operations. The complexity of managing multiple VAT rates raises administrative costs for both the government and businesses. These varying rates can lead to skewed consumer decisions as price comparisons become more challenging, impacting overall market dynamics. Mirrlees [40] highlights that these distortions and administrative burdens can slow economic growth. Alternative approaches, such as broadening the VAT base and applying a uniform rate, could simplify the system and reduce inefficiencies. However, evidence from Ormaechea et al. [38] suggests that increasing VAT rates with a standard rate might not always improve economic outcomes. Furthermore, Lima et al. [41] indicate that higher VAT rates can negatively affect short-term economic output. Nevertheless, sales performance across industries can fluctuate widely, with some experiencing growth while others facing declines. Analysis of financial indicators in alignment with IMF programs, which often involve varied tax revenues over three years, shows a correlation with improved outcomes. Reinsberg et al. [42] note that IMF programs increase goods and services taxation by 0.7 % of GDP and yield positive results. However, the global financial crisis of 2007–2009 appears to have triggered a shift in economic growth patterns in developed nations. From 1993 to 2007, the median GDP per capita growth rate in OECD countries was 2.5 %, but it fell to 1.51 % from 2010 to 2018, reflecting a slowdown. Summers et al. [43] attribute this decline to secular stagnation, a theory suggesting that long-term growth is impeded by structural factors within the economy. The relationship between VAT policies and economic outcomes varies across contexts, as observed in several cases. In China, a 1 % reduction in export VAT correlates with a substantial 7.2 % increase in the value of permitted exports, indicating that lower VAT rates can significantly enhance export performance [44]. Similarly, transitioning from a business tax to a VAT encourages innovation by motivating companies to improve their output [45]. In Indonesia, the implementation of electronic invoices and enhanced taxpayer compliance have positively influenced VAT revenue, highlighting the effectiveness of improved administrative practices [46]. In South Korea, the level of trust in the VAT system and associated incentives, such as higher discounts, directly affect compliance rates, suggesting that trust and motivational factors play a crucial role [47]. Across Asia, implicit tariffs and government efficiency positively influence VAT revenue, whereas increased import volumes can diminish it, underscoring the importance of effective government practices and services in optimizing VAT revenue while managing import impacts.

In contrast, current findings on VAT receipts reveal that research by Permadi et al. [48] indicates that factors such as standard rates, fiscal shortages, effectiveness, control of corruption, the rule of law, and democracy have minimal impact on VAT receipts. In contrast, previous studies, including [49], highlight that changes in VAT can significantly affect business performance, though the anticipated benefits of VAT are not always realized. In Saudi Arabia, the impact of VAT on economic output may vary based on the country's development level, necessitating a careful assessment by the government before implementing tax rate reforms. Investing in robust administrative infrastructure to improve VAT collection and compliance is recommended for GCC countries contemplating VAT introduction or reform. Effective tax administration and enforcement mechanisms can help minimize tax evasion and maximize VAT revenue. Additionally, providing comprehensive training and support for businesses on VAT regulations can enhance compliance and reduce errors, contributing to a more efficient VAT system overall.

### 6.5 Managerial and policy implications

The findings of this study demonstrate the critical need for non-financial companies to adopt enhanced financial management practices in response to the VAT increase. The observed decline in Total Assets (TA) and Shareholders' Equity (SE) indicates a potential erosion of company value, necessitating more rigorous asset management and capital allocation strategies. Managers should consider implementing advanced financial planning tools, such as scenario analysis and stress testing, to better anticipate and mitigate the impact of future tax changes. Additionally, the reduction in Net Income (NI) highlights the importance of optimizing cost structures. Companies may benefit from conducting detailed cost-benefit analyses to identify areas where efficiency gains can be realized, particularly in operational processes. This could involve deploying automation technologies and lean management techniques to streamline operations and reduce overhead costs. Moreover, the decrease in Cash at the End of the Period (CEP) further underscores the importance of liquidity management in maintaining financial stability under increased tax pressure. Managers should prioritize maintaining an optimal level of working capital by implementing robust cash flow forecasting models that incorporate the effects of tax liabilities. Moreover, companies may need to explore alternative financing options, such as revolving credit facilities or strategic partnerships, to ensure sufficient liquidity during periods of fiscal tightening. Diversification of revenue streams through geographic expansion or product innovation should also be considered to buffer against revenue volatility induced by tax policy changes. These strategies collectively enhance the company's resilience, enabling it to withstand better the financial pressures imposed by VAT increases.

Furthermore, the study's results significantly affect tax policy design, particularly in emerging markets where the corporate sector's ability to absorb tax shocks is often limited. The substantial impact of the VAT increase on corporate financial health suggests that policymakers should adopt a more nuanced approach to tax reform. Policymakers must consider the elasticity of demand in different sectors when setting tax rates, as higher VAT rates may disproportionately affect sectors with lower pricing power, leading to reduced profitability and potential market distortions. A phased or tiered VAT implementation could be considered to balance revenue generation with economic stability, allowing companies time to adjust their pricing and operational strategies. In addition, introducing complementary tax relief measures, such as accelerated depreciation allowances or investment tax credits, could mitigate the negative impact on capital investment. Policymakers should also explore the possibility of sector-specific VAT exemptions or reduced rates for industries particularly vulnerable to economic fluctuations. This would help maintain the competitive edge of key sectors while still achieving overall revenue objectives. Furthermore, establishing a transparent and consistent tax policy framework is crucial. Policymakers should continuously dialogue with industry stakeholders to assess tax policy's real-time impact and make necessary data-driven adjustments. This collaborative approach would ensure that VAT policies are aligned with broader economic goals, such as promoting investment, innovation, and sustainable growth while minimizing unintended negative consequences.

### 6.6 Limitations and future works

One major limitation of this study is its small sample size, focusing exclusively on non-financial listed companies in Saudi Arabia. This narrow scope may not comprehensively represent the entire market or capture the diverse impacts across different sectors. Additionally, the study concentrates solely on the effects of VAT, neglecting other types of taxes that could also influence corporate financial performance. This limited focus restricts the ability to draw broader conclusions about taxation. The time frame of the analysis, covering only 2019 and 2020, is another constraint. This period is significantly influenced by the economic disruptions caused by the COVID-19 pandemic, which complicates the isolation of VAT effects from those of the pandemic. Moreover, the geographical limitation to Saudi Arabia means the findings may not be readily generalizable to other countries. The study also assumes a well-implemented VAT system, which may not reflect the implementation and efficiency in other developing nations.

Future research should address these limitations by incorporating a broader tax analysis. Including various types of taxes would provide a more holistic understanding of how taxation impacts corporate performance. Extending the study's time frame would help distinguish the effects of VAT from temporary economic shocks like the COVID-19 pandemic. Furthermore, conducting cross-country studies, particularly within the GCC and other developing regions, would enhance the generalizability and applicability of the findings. Moreover, the sector-specific analysis could also be valuable, as different industries may experience varying impacts from VAT changes. Investigating the effects of VAT on the informal economy and tax evasion in developing countries would offer deeper insights into the broader economic implications. Furthermore, more detailed studies on the relationship between taxation and governance in developing countries are needed to explore this complex interaction comprehensively. Methodologically, future research could benefit from employing advanced analytical techniques such as meta-analysis and probability charts to improve the robustness of findings. Lastly, focusing on the policy implications and adjustments necessary for optimizing VAT systems, especially in the post-pandemic context, would provide practical recommendations for policymakers aiming to enhance economic outcomes through tax reforms.

## 7 Conclusion

This study provides a comprehensive analysis of the impact of the 15 % Value Added Tax (VAT) increase on the financial performance of non-financial listed companies in Saudi Arabia. Utilizing the Event Study Method (ESM) alongside the Autoregressive Integrated Moving Average (ARIMA) model, the research focuses on critical financial indicators such as Shareholders' Equity, Total Income, Total Revenues, Net Income, Total Expenses, and Cash at the End of the Period. The data revealed a notable decline in financial performance following the VAT increase, with Shareholders' Equity decreasing by an average of 12 % and total Revenues and Net Income dropping by 8 % and 10 %, respectively. In comparison, Total Expenses rose by 6 %, and Cash at the End of the Period declined by 7 %. These findings indicate that the VAT hike significantly strained the companies' financial performance, impacting their profitability and cash flow. This study uniquely contributes to VAT and taxation literature by examining the effects of VAT on non-financial companies in an emerging market such as Saudi Arabia, filling a critical gap in existing research. Using detailed disaggregated data and advanced analytical methods establishes robust correlations over two distinct periods, providing a nuanced understanding of the VAT's impact. The empirical findings suggest that while the VAT increase aims to enhance government revenues, it imposes substantial financial strain on companies, potentially leading to long-term economic repercussions. Policymakers are encouraged to consider these implications carefully and implement measures to optimize the VAT system, supporting business resilience and ensuring sustainable economic growth. Additionally, this research highlights the necessity for refining VAT policies to encourage sustainable economic growth and reduce regional economic disparities. The study provides valuable insights for policymakers, suggesting that careful policy adjustments are needed to optimize VAT systems, particularly in the post-pandemic context. These findings underscore the importance of rigorous analysis and thoughtful policy design to balance fiscal objectives with economic stability in taxation reform.

It must be noted that this study primarily focuses on the immediate financial impacts of the VAT increase by examining 11 key financial indicators. However, we acknowledge that the relationship between financial performance and the variables that condition it, particularly in the context of the VAT increase, is complex. Introducing additional variables—such as market size, sector-specific factors, or external economic conditions—could have provided a more comprehensive understanding of this relationship. Future research could enrich this analysis by incorporating these broader factors to offer deeper insights into how VAT impacts financial performance in varying contexts.

## CRediT authorship contribution statement

**Mohammed Ibrahim Al-Otaibi:** Writing – original draft, Validation, Software, Resources, Methodology, Investigation, Formal analysis, Data curation, Conceptualization. **Normaziah Mohd Nor:** Writing – review & editing, Supervision, Resources, Project administration, Conceptualization. **Yusniyati Yusri:** Writing – review & editing, Supervision, Project administration, Conceptualization. **Nedaa Guzaiz:** Writing – review & editing, Validation, Formal analysis, Conceptualization.

## Data availability statement

All relevant data are included in the paper.

## Funding

Not Applicable.

## Declaration of competing interest

The authors declare that they have no known competing financial interests or personal relationships that could have appeared to influence the work reported in this paper.
